# Analysis of transporter associated with antigen presentation (*TAP*) genes polymorphisms with HIV-1 infection

**DOI:** 10.1007/s11010-019-03649-x

**Published:** 2019-11-16

**Authors:** Abaineh Munshea Abitew, Ranbir Chander Sobti, Vijay Lakshmi Sharma, Ajay Wanchu

**Affiliations:** 1 Biotechnology Research Institute, Bahir Dar University, P.O. Box 79, Bahir Dar, Ethiopia; 2 Department of Biotechnology, Panjab University, Chandigarh, India; 3 Department of Zoology, Panjab University, Chandigarh, India; 4 Department of Internal Medicine, Postgraduate Institute of Medical Education and Research, Chandigarh, India

**Keywords:** HIV-1, HLA, PCR- RFLP, Polymorphisms, *TAP1* and *TAP2* genes

## Abstract

Human leukocyte antigen (HLA) class I molecules of the human major histocompatibility complex (MHC) play an important role in modulating immune response. HLA class I molecules present antigenic peptides to CD8^**+**^ T cells and thereby play a role in the immune surveillance of cells infected with viruses. *TAP1* and *TAP2* are *MHC*-*II*-encoded genes necessary for the generation of a cellular immune response and polymorphism of these genes can influence the specificity of peptides preferentially presented by the MHC class I molecules and the outcome of the immune response. Several studies implicated genetic variation in *TAP* genes to various immune-mediated and infectious diseases. To determine the correlation between HIV-1 infection and the *TAP1* and *TAP2* genes polymorphisms, we performed PCR–RFLP assay of these genes in 500 HIV-1 seropositives and the matched seronegative individuals. Statistical analysis of the data disclosed no correlation between *TAP1* (*C/T intron 7*) gene polymorphism and HIV-1/AIDS disease. However, the current results demonstrated that the heterozygous *A/G* [OR (95% CI) 1.39 (1.06–1.83), *P* = 0.0171] and homozygous *G/G* [OR (95% CI) 3.38(1.56–7.46), *P* = 0.0010] variants of *TAP2* (*A/G exon 11*) (*T665A*) gene are positively associated with an increased risk of HIV-1/AIDS infection. This case–control analysis might suggest a possible role of *TAP2* (*A/G exon 11*) (*T665A*) gene in the susceptibility to HIV-1 infection and disease outcome among North Indian patients.

## Introduction

The human immunodeficiency virus infection (HIV) is essentially an infection of the immune system, with progressive and profound defects in the cell-mediated immune response, in which viral and host genetic factors may have an important role [1, 2]. The host genetic variations play critical roles in influencing an individual’s infectiousness by facilitating viral cell entry and modulating immune responses. The major host genetic factors those have been reported to be linked to the HIV-1 infection and disease progression include the variants of human leukocyte antigen (*HLA*) alleles and HIV-1 co-receptors and their respective chemokine ligands [3–5].

*HLA class I* molecules play a major role in the immune response against viral infections and transformed cells by presenting peptide antigens to cytotoxic T lymphocytes (CTL). CTLs are major effectors in the cellular arm of antigen-specific immune responses. They play a major role in protection and recovery from viral infection, mediate allograft rejection, are implicated in certain autoimmune diseases, and contribute to protection and recovery from certain bacterial and parasitic infections and to tumor immunity. The recognition and subsequent lysis of virus-infected target-host cells by CTL requires mediation by *HLA class I* molecules loaded with viral antigen-derived peptides on the cell surface. This HLA class I antigen-peptide complex is transported to the cell surface and is recognized by CTLs as a signal of virus infection, cancer, or autoimmune disease and destroy the diseased cell [6, 7].

Transporter associated with antigen processing (TAP) protein complex belongs to the ATP-binding-cassette transporter family, a number of which are associated with serious human diseases. TAP is interferon-γ (*IFN*-*γ*) inducible and essential for peptide delivery from the cytosol into the lumen of the endoplasmic reticulum (ER), where these peptides are loaded on major histocompatibility complex (MHC) I molecules [8]. Loaded MHC-I molecules leave the ER and display their antigenic cargo on the cell surface to cytotoxic T cells. Subsequently, virus-infected or malignantly transformed cells can be eliminated. The functional TAP complex is a heterodimer of TAP1 and TAP2, each subunit containing transmembrane domains and an ATP-binding motif and their function is dependent on hydrolysis of adenosine triphosphate (ATP) [8, 9].

TAP proteins are encoded by *TAP1* and *TAP2* genes, a cluster of genes located within the HLA class II region of the *HLA* between the *HLA*- *DP* and -*DQ* loci on the short arm of human chromosome 6 [10]. *TAP1* and *TAP2* genes comprise 8–12 kilobase pairs and consist of 11 exons each. Eight of the 11 exons are of the same length, and the remaining 3 exons differ in length by 100 (exon 1), 3 (exon 9), and 78 (exon 11) nucleotides [11]. The *TAP1* and *TAP2* genes are polymorphic; seven *TAP1* alleles and four *TAP2* alleles have been officially named by WHO HLA Nomenclature Committee [12]. Single-nucleotide polymorphism in these genes may result in structural changes that prevent TAP heterodimer formation and alter antigen recognition and presentation [8], which may lead to low or no expression of MHC-I molecules on the cell surface and consequently an adverse affect in the immune response.

*TAP1* and *TAP2* are essential in assembly and expression of *HLA class I* proteins for subsequent generation of CTL-mediated cellular immune response against viral infection and transformed cells. Genetic polymorphism of these genes could influence the specificity of peptides preferentially presented by the HLA class I molecules and the outcome of the immune response. Deleterious mutations or dysregulation of *TAP* genes invariably disrupts or blocks the class I antigen-presenting pathway, as evidenced in several experimental models, human case studies, and malignancies [13]. Neither TAP1 nor TAP2 will be capable of binding the peptides alone [14]. Expression of HLA class I molecules is inhibited if either of the two transporters of antigenic peptides 1 and 2 (TAP1/2) is absent [15].

The TAP genes are known to be polymorphic with several mutations in different populations. There is clear evidence that these polymorphisms can affect the functionality of the transporters and serve as markers for human disease. The *TAP* genes have been reported to be important candidates for disease susceptibility in view of the fact that these genes are located within the *HLA class II* region between HLA-DP and –DQ loci, play an important role in endogenous antigen processing, and have functional relation with *HLA* molecules and polymorphisms. Evidences supporting their role in inflammatory/immune-mediated, viral, and bacterial diseases and various types of cancers have been well documented [16–22].

Single-nucleotide polymorphisms in *TAP1*(***C****/T intron 7*) and *TAP2* (*A/G exon 11*) (T665A) genes which we considered in our study have been associated with various diseases, such as idiopathic bronchiectasis [23], hematological malignancies [24], colorectal cancer [25], and the development of allergic rhinitis [26]. Hence, it is plausible to suggest these *TAP* genes might also be associated with the risk of HIV-1 infection and disease outcome. In fact, earlier studies of Kaslow et al. [27] demonstrated that the combinations of *TAP* genes along with particular HLA class I or II alleles may influence the period from infection to the onset of AIDS in human immunodeficiency virus infection. Moreover, Liu et al. [28] demonstrated the significant association of the *TAP2 Ala665* variant with resistance to HIV-1 infection and the borderline significant association with *TAPI Gly637.*

Because of their noticeable impact on the various immune-mediated and infectious diseases, we hypothesized that polymorphisms in *TAP* genes might also be correlated with the risk and outcomes of HIV-1 infection. With this perspective, we performed genotyping analyses to test the hypothesis for these single-nucleotide polymorphisms (SNPs) in HIV-1/AIDS seropositive patients and healthy controls from a north Indian population. The present study set out with the aim of examining the distribution of SNPs in the two MHC-II encoded *TAP1 and TAPS 2* genes in North Indians and evaluating whether polymorphisms in these genes are associated with the risk of HIV-1 infection and disease outcome when compared with the controls.

## Materials and methods

### Study population and sample collection

The study population consisted of five hundred seropositive HIV-1/AIDS patients and an equal number of age- and gender-matched seronegative healthy controls from the North Indian population. All patients included in this study were follow-up and newly diagnosed cases in the Immunodeficiency Clinic of the Department of Internal Medicine at the Postgraduate Institute of Medical Education and Research (PGIMER) Chandigarh, India. Of the 500 cases, 174 were females and 326 were males with mean age of 35.4 ± 7.9, while that of controls 183 were females and 317 were males with mean age of 36.2 ± 9.8 years.

Information about age, sex, occupation, and other relevant clinical data was gathered from the immunological card of the study subjects. 2–3 mL of peripheral blood sample was drawn from all of the study subjects and collected in an EDTA-coated tube for genomic DNA extraction. Then, the samples were kept at – 80 °C freezer until used for DNA isolation.

### Genomic DNA extraction

Genomic DNA of each subject was extracted from peripheral blood mononuclear leukocytes (PBLCs) by sodium dodecyl sulfate (SDS) lysis and proteinase K digestion followed by standard phenol–chloroform methods according to standard protocol as previously described [29] and the quality of the extracts was verified using 1% agarose gel electrophoresis. The genomic DNA samples were then genotyped for detecting SNPs of the *TAP1*(*C/T intron 7*) and *TAP2* (*A/G exon 11*) using PCR–RFLP assay.

### TAP genotyping by PCR–RFLP Method

SNPs at *TAP1* (*C/T intron 7*) and *TAP*2 (*A/G exon 11*) (T665A) were assayed by polymerase chain reaction-restriction fragment length polymorphism (PCR–RFLP) as described previously [30] with some modification. Briefly, PCRs for TAP1 were performed in a total reaction volume of 25 μl containing 2 μl of genomic DNA, 2.5 μl PCR buffer, 2.5 μl BSA, 1.5 μl MgCl2, 0.125 μl dNTP mix, 0.5 μl of each primer (Forward: 5′-GTG CTC TCA CGT TCC AAG GA-3′, Reverse: 5′-AGG AGT AGA GAT AGA AGA ACC-3′), and 1.5 U Taq polymerase under the following PCR conditions: a denaturation step for 2 min at 94 °C followed by 35 cycles of a denaturation for 30 s at 94 °C, annealing for 30 s at 55 °C, extension for 1 min at 72 °C, and a final extension for 7 min at 72 °C. Likewise, the PCRs for TAP2 were also carried out in a total reaction volume of 25 μl containing 2 μl of genomic DNA, 2.5 μl PCR buffer, 2.5 μl BSA, 1.5 μl MgCl2, 0.125 μl dNTP, 0.5 μl of each primer (Forward: 5′- GGT GAT TGC TCA CAG GCT GCC G-3′ Reverse: 5′-CAC AGC TCT AGG GAA ACT C-3′) and 1 U Taq polymerase under the following conditions: a denaturation step for 2 min at 94 °C followed by 35 cycles of a denaturation for 30 s at 94 °C, annealing for 30 s at 61 °C, extension for 1 min at 72 °C and a final extension for 7 min at 72 °C. All reactions were carried out in a Bio-Rad **My**Cycler.

The amplified 183- and 225-bp PCR products of TAP1 and TAP2, respectively, were verified by using 2% agarose gel after electrophoresis and ethidium bromide staining. The remaining PCR products were digested with *Msp1* restriction endonuclease (New England Biolabs). Genotypes of both genes were determined by RFLP analysis of the PCR products. The restriction assay was carried out in a total volume of 15 μl containing 10 μl of PCR product, five units of restriction enzyme, 1.5 µL of 10 × buffers, and 3.5 μl of distilled water. The restriction digestion mixture was then incubated at 37 °C for overnight. Then, all of the generated fragments ware analyzed by electrophoresis on ethidium bromide-stained 3% agarose gel and visualized in UV light.

### Statistical analysis

For statistical analysis of the data, we used SPSS software, version 11.5 (SPSS Inc., Chicago, IL) for windows. Allelic and genotype frequencies of *TAP1*(*C/T intron 7*) and *TAP2* (*A/G exon 11*) genes were estimated by direct manual counts and expressed as percentages. Comparison between patients and controls was done using 2 × 2 contingency tables and Chi square (*χ*^2^) test with Yates correction (StatCalc programme, Epi Info Version 6.04, CDC, Atlanta, GA, USA). The frequencies were also tested for Hardy–Weinberg equilibrium by calculating allele frequencies using Pearson’s *χ*^2^ test with one degree of freedom. Throughout this study, a *p* value of < 0.05 was considered statistically significant.

### Ethical consideration

The study protocol was reviewed and approved by the institutional ethical committee of PGIMER. A written informed consent was obtained from both voluntary HIV/AIDS patients and healthy controls. Those who refused to be part of the study were excluded.

## Results

### Characteristics of the study population

A total of 500 cases and an equal number of age- and sex-matched controls participated in this case control study. The demographics of the cases and controls enrolled in this study are shown in Table 1. The percentage of male subjects was higher than for females in both cases and controls; however, there were no significant difference in gender distribution between cases and controls (65.2% vs. 63.4% male and 34.8% vs. 36.6% female, *P* = 0.553). The mean age ± SD years was 35.4 ± 7.9 years for cases and 36.2 ± 9.8 years for controls. There was no significant difference in mean age of cases and the controls (*P* = 0.180).

**Table 1:** Demographic data of patients and controls used in this study

Variables	HIV-1/AIDS patients	Healthy controls	*P* value
Number	*N* = 500	*N* = 500	
Gender *N* (%)			
Male	326 (65.2%)	317 (63.4%)	0.553
Female	174 (34.8%)	183 (36.6%)	
Mean age (years) Mean ± SD	35.39 ± 7.90	36.15 ± 9.75	0.180

### *TAP1* (*C/T intron 7*) *and TAP2* (*A/G exon 11*) **(T665A)***polymorphism analysis by PCR*–*RFLP*

**Fig. 1:**
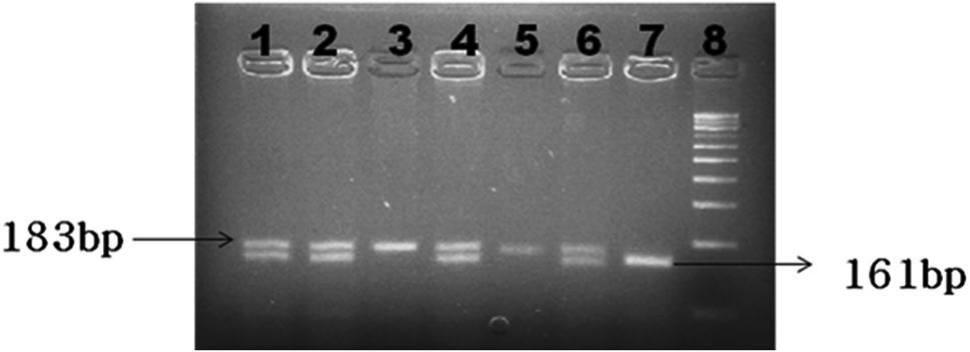
Representative agarose gel picture of *TAP1*gene after digestion with *Msp I* restriction endonuclease. Lanes 1, 2, 4, and 6 represent heterozygous C/T genotype. Lanes 3 and 5 represent homozygous wild C/C genotype. Lane 7 represents homozygous variant T/T genotype. Lane 8 represents 100-bp DNA marker

**Fig. 2:**
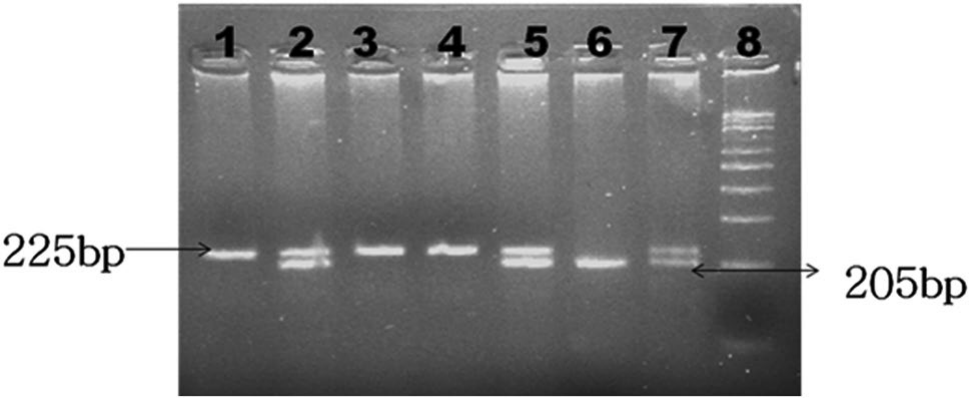
Representative agarose gel picture of *TAP2* gene after digestion with *Msp I* restriction endonuclease. Lanes 1, 3, and 4 represent homozygous wild A/A genotype. Lanes 2, 5, and 7 represent heterozygous A/G genotype. Lane 6 represents homozygous variant G/G genotype. Lane 8 represents 100-bp DNA marker

After *MspI* digestion of the PCR products of *TAP1* gene, individuals homozygous for Wild C/C genotype were identified by the presence of only 183-bp fragments, heterozygous for C/T genotype by the presence of 183-, 161-, and 22-bp fragments, and homozygous variant for T/T genotype by 161- and 22-bp fragments (Fig. 1). While, in case of TAP2 with the same restriction assay condition, A/A genotype was identified by the presence of only 225-bp fragment, A/G genotype was identified by the presence of 225-, 205-, and 20-bp fragments and G/G genotype by 205- and 20-bp fragments (Fig. 2).

### The prevalence of genotypes of the TAP1 (C/T intron 7) and TAP2 (A/G exon 11) **(T665A)** genes polymorphisms and risk of HIV-1 infection

In the study groups comprising 500 HIV-1/AIDS patients (study group 1) and 500 healthy controls (study group 2), we detected two previously described SNPs: *TAP1* (*C/T intron 7*) and *TAP2* (*A/G exon 11*) (T665A). The allelic frequencies and distributions of genotypes of polymorphisms in *TAP1*and *TAP2* genes between HIV-1/AIDS patients and control subjects are summarized in Table 2. Comparing cases to controls, the prevalence of the *C/C*, *C/T,* and *T/T* genotypes of *TAP1* (C/T intron) gene polymorphism in cases was 39.0, 55.2, and 5.8%, respectively, against 41.6, 53.8, and 4.6%. The proportions of C and T alleles in cases were 66.6 and 33.4% while in controls were 68.5 and 31.5%, respectively, (Table 2). The frequencies of the *A/A, A/G,* and *G/G* genotypes of *TAP2* (A/G exon 11) (T665A) polymorphism in HIV-1 patients were 30.4, 63.8, and 5.8%, respectively. While the percentages of *A/A, A/G* and *G/G* genotypes were 39.0%, 58.8% and 2.2% respectively in healthy controls. The proportions of *A* and *G* alleles in cases were 62.3 and 37.7% while in controls were 68.4 and 31.6%, respectively (Table 2). The distribution of each of genotypic variants met the conditions of Hardy–Weinberg equilibrium.

**Table 2:** Genotype distribution of *TAP1 and TAP2* genes among the study groups

	Cases (*N* = 500) (%)	Controls (*N* = 500) (%)	OR(95% CI)	*P* value
*TAP1 C/T intron genotype distribution and allelic frequencies*				
*CC*	195 (39.0)	208 (41.6)	1.00 Ref	
*CT*	276 (55.2)	269 (53.8)	1.09 (0.84–1.43)	0.5340
*TT*	29 (5.8)	23 (4.6)	1.34 (0.72–2.50)	0.3920
*CT *+* TT*	305 (61.0)	292 (58.4)	1.11 (0.86–1.45)	0.4390
*C allele*	666 (66.6)	685 (68.5)	1.00 Ref	
*T allele*	334 (33.4)	315 (31.5)	1.09 (0.90–1.32)	0.3890
*TAP2 A/G exon 11*((T665A)) *genotype distribution and allelic frequencies*				
*AA*	152 (30.4)	195 (39.0)	1.00 Ref	
*AG*	319 (63.8)	294 (58.8)	1.39 (1.06–1.83)	0.0171
*GG*	29 (5.8)	11 (2.2)	3.38 (1.56–7.46)	0.0010
*A allele*	623 (62.3)	684 (68.4)	1.00 Ref	
*G allele*	377 (37.7)	316 (31.6)	1.31 (1.08–1.58)	0.0048

OR odds ratio, OR was calculated by Epi info. Version 3.5.1. (Center for disease control and prevention), CI 95% confidence interval, *P* value ≤ 0.05 significant

There was no significant difference in the distribution of the genotypes and alleles of *TAP1* (C/T intron) gene polymorphism in both groups of the study subjects. The frequency of heterozygous *C/T* genotype did not show statistically significant difference in the patients with HIV-1 infection in comparison with the frequency observed in the controls [OR (95% CI) 1.09 (0.84–1.43), *P* = 0.534]. Similarly odds ratio for homozygote variant *T/T* genotype (OR (95% CI) 1.34 (0.72–2.50) showed no significant difference between patients and control groups (*P* = 0.392).

In contrast to *TAP1* (C/T intron), statistically significant differences were detected in the distribution of genotypes and allele of *TAP2* (*A/G exon 11*) (T665A) between the two study groups. The frequency of heterozygous *A/G* genotype was significantly higher in patients with HIV-1/AIDS, compared to those in the controls [OR (95% CI) 1.39 (1.06–1.83), *P* = 0.0171]. The frequency of homozygous variant *G/G* was also remarkably higher in patients than controls, [OR (95% CI): 3.38 (1.56–7.46), *P* = 0.0010].

The heterozygous *A/G* and the homozygous variant *G/G* genotypes of SNPs in *TAP2* (A/G exon 11) (T665A) gene polymorphism investigated in this study were found to be strongly associated with risk of HIV-1 infection. On the other hand, no significant *TAP1*(C/T intron 7) gene association with the risk of (resistance/susceptibility) to HIV-1 infection was identified. The present result suggested that individuals possessing the variant *G allele* of TAP2 (A/G exon 11) (T665A) had an elevated risk of HIV-1 infection as the large proportion of HIV-positive individuals carried this variant as compared to HIV-1 seronegative groups.

## Discussion

The human immunodeficiency virus infection (HIV) is essentially a disease of the immune system. If left untreated, HIV infection results in a progressive loss of the CD4^+^ T helper cells, leading to severe immunosuppression that predisposes a patient to wide range of opportunistic infections, malignant neoplasm, and neurological complications that rarely occur in persons with intact immune function [31, 32].There are sufficient evidences suggesting susceptibility or resistance to infection with HIV-1 as well as the subsequent rate of disease progression to AIDS is multifactorial, including viral, host genetic, and immunological factors. In general, genetic variations in human population exert a major influence on susceptibility and progression of infectious diseases [33]. In context of HIV/AIDS infection, several prior studies examined the role of host genes in the course of HIV-1 infection in different populations and among all major risk groups. Such investigations have revealed that genetic polymorphisms in chemokine receptors or in their natural ligands are likely to influence susceptibility or resistance to HIV-1 infection as well as the subsequent rate of disease progression to AIDS [3, 34]. Furthermore, genes within human leukocyte antigens (HLA) that regulate host immune response to infection have been correlated with the clinical course of HIV-1 infection [35].

The recognition of virally infected cells and the subsequent immune surveillance by CD8^+^ T cells is dependent upon a complex series of degradation, transport, and association reactions that permit peptide fragments of endogenously synthesized viral proteins to be displayed on the surfaces of infected cells in association with class I MHC molecules [36–38]. *TAP1* and *TAP*2 are IFN-γ-inducible cluster of genes within HLA class II region on the short arm of chromosome 6 [10]. They are necessary for generation of CD8^+^-mediated cellular immune response against viral infections and genetic polymorphisms of these genes can influence the specificity of antigenic peptides preferentially presented by the HLA class I molecules and the outcome of infection [8, 9].

Several reports have described genetic associations of *TAP1* and *TAP2* polymorphisms with autoimmune/inflammatory diseases including systemic lupus erythematosus [16], hypersensitivity pneumonitis [19], vitiligo [39], Graves’ disease [40], type I diabetes [41], systemic sclerosis [42], and ankylosing spondylitis [43].

Furthermore, investigations by various researchers also revealed the correlation of *TAP* genes with the immune response to infectious diseases such as tuberculosis [17], hepatitis C virus [22], hepatitis B virus [18, 44], and dengue viral infection [45]. Such associations of *TAP* genes with various immune-mediated, infectious diseases and transformed cells have been attributed to the location of these genes within the *HLA class II* region between HLA-DP and –DQ loci, their role in endogenous antigen processing, and functional relation with *HLA* molecules and their polymorphisms. In context of HIV/AIDS and *TAP gene* polymorphisms, earlier studies of Kaslow et al. [27] and Keet et al. [46] demonstrated the influence of certain variants of *TAP* genes along with particular *HLA class I* or *II* alleles on the period from infection to the onset of AIDS in HIV infection. Besides, Liu et al. [28] found a significant correlation of the *TAP2 Ala665* variant with resistance to HIV-1 infection and borderline significant association with *the TAP1 Gly637.*

To our knowledge, the present study is the first to examine the genetic polymorphisms of *TAP1* (*C/T intron 7*) *and TAP2* (*A/G exon 11*) (T665A) in the HIV-1/AIDS patients and controls from diverse ethnic groups from North India. There was no significant difference in the distribution of the alleles and genotypes of *TAP1* (*C/T intron 7*) gene polymorphism in both groups of the study subjects. However, statistically significant difference was detected in the distribution of allele and genotypes of *TAP2* (*A/G* exon 11) (T665A) between the two study groups. We didn’t find any evidence of association between *TAP1* gene polymorphism and the risk of HIV-1 infection whereas individuals carrying heterozygous (*A/G*) and homozygous variant (*G/G*) genotypes of *TAP2* gene polymorphism were at 1.39- and 3.38-folds of increased risk of HIV-1 infection and disease outcome, respectively, than those of carrying homozygous wild (*A/A*) genotype.

Viral infection induces CD8^+^ cytotoxic T lymphocytes (CTL) responses by presenting viral antigen on *HLA class I* molecules at the surface of infected cells [47]. As to HIV infection, the majority of *HLA class I*-restricted epitopes in the HIV-env proteins are generated by TAP1- and TAP2-dependent mechanisms [48]. The HIV-1 env-specific CD8^**+**^ T cells have been shown to be an important component of the initial cellular immune response to acute HIV-1 infection as they control viral replication and thereby influence the course of disease development [38, 49]. The influence of genetic polymorphisms in *TAP* genes on the specificity of peptides preferentially presented by the HLA class I molecules and the outcome of the immune response has been well documented [13]. Therefore, the results of the present study suggests polymorphism of *TAP2* gene, which plays a pivotal role in HLA class I antigen-presenting pathway might be associated with increased risk of HIV-1 infection among seropositive North Indians. Subjects with *A/G* and *G/G* genotypes are more susceptible to HIV-1 infection and disease outcome than those with *A/A* genotype. Furthermore, though we didn’t find any evidence of association between *TAP1* gene polymorphism and the risk of HIV-1 infection, we can’t entirely exclude the role of this immunomodulatory gene in the outcome of HIV-1 infection because of its role in facilitating HLA-I-mediated antigen-presenting pathway resulting CD8^+^ T cell-mediated immune surveillance against viral infections.

In summary, results of this study showed significant association of the genetic variants of *TAP2* (*A/G exon 11*) (T665A) gene polymorphism with increased risk of HIV-1 infection. These results suggest the inheritance of the *TAP* genes might contribute to the disease susceptibility and progression to HIV-1/AIDS. However, further studies, which take other polymorphic sites of *TAP* genes into account, need to be undertaken in order to understand the association between *TAP* genes polymorphisms and HIV-1/AIDS and reach at definite remarks on the findings of the present study. Since the regulation of immune response is a complicated process involving numerous genes, it would be expected that individual gene might only have limited effect to disease susceptibility.
